# Supplementary material for: Broadband nonlinear modulation of incoherent light using a transparent optoelectronic neuron array
Source: Nat Commun. 2024 Mar 18;15:2433. doi: 10.1038/s41467-024-46387-5 (PMC10948843; doi:10.1038/s41467-024-46387-5)
Supplement: Supplementary file 1 — Supplementary Information [file 41467_2024_46387_MOESM1_ESM.pdf]

Broadband nonlinear modulation of incoherent light using a transparent optoelectronic neuron array

Dehui Zhang[1], Dong Xu[2], Yuhang Li[3], Yi Luo[3], Jingtian Hu[3], Jingxuan Zhou[2], Yucheng Zhang[2], Boxuan Zhou[2], Peiqi Wang[1], Xurong Li[3], Bijie Bai[3], Huaying Ren[1], Laiyuan Wang[1], Ao Zhang[2], Mona Jarrahi[3,4], Yu Huang[2,4], Aydogan Ozcan[3,4*], and Xiangfeng Duan[1,4,*]

Table of contents

Supplementary Figures 1-8	Pages 2-9
Supplementary Tables 1, 2	Pages 10-11
Supplementary Video 1	Page 12
Supplementary Note 1	Page 13-15
Supplementary References	Page 16

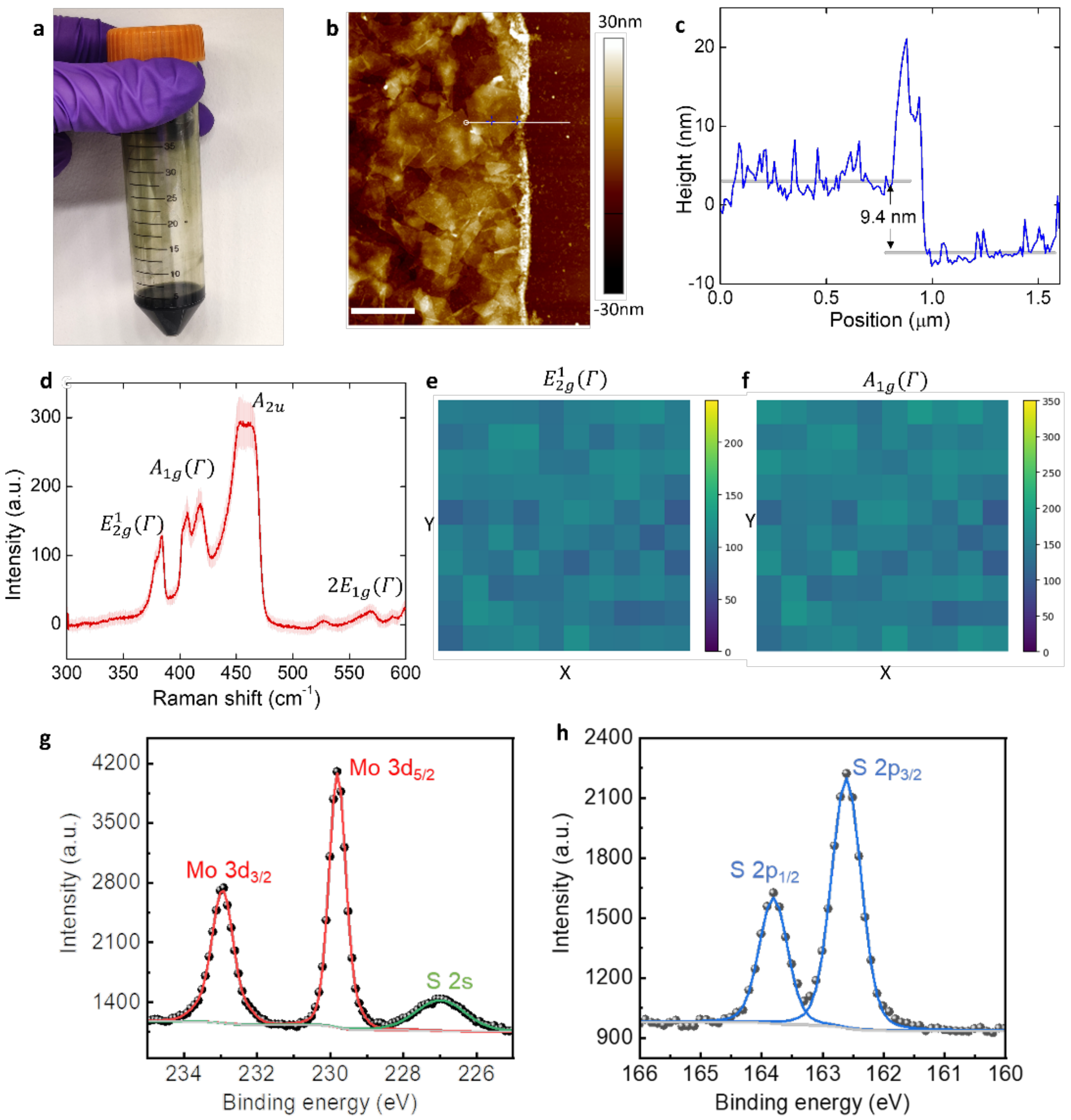

Supplementary Fig. 1 | Material characterization of solution-processed MoS_2 van der Waals thin film (VDWTF). **a**. The as-fabricated MoS_2 ink with nanosheets dispersed in IPA. **b**. The AFM measurement on the VDWTF. Scale bar: 1 um. **c**. The height profile along the thin white cutline in **b**, with a thickness around 9.4 nm. **d**. The Raman spectra of the VDWTF with an excitation wavelength of 633 nm. Solid line: mean value of 10-by-10 sampling points over a region of 1 mm. The error bars are defined with the standard deviation over 100 sampling points. **e**. The $E^1_{2g}(\Gamma)$ peak intensity distribution over the 100 sampling points, with inter-pixel spacings at 100 μm. **f**. The $A_{1g}(\Gamma)$ peak intensity distribution. **g**, **h**. Core level XPS spectra of MoS_2 VDWTF with fitting for Mo 3d (red), S 2s (green), and S 2p (blue), respectively. The stoichiometric molar ratio Mo : S is 1 : 2.02, close to the theoretical expectation within the measurement error, suggesting satisfactory crystal quality and a low defect density.

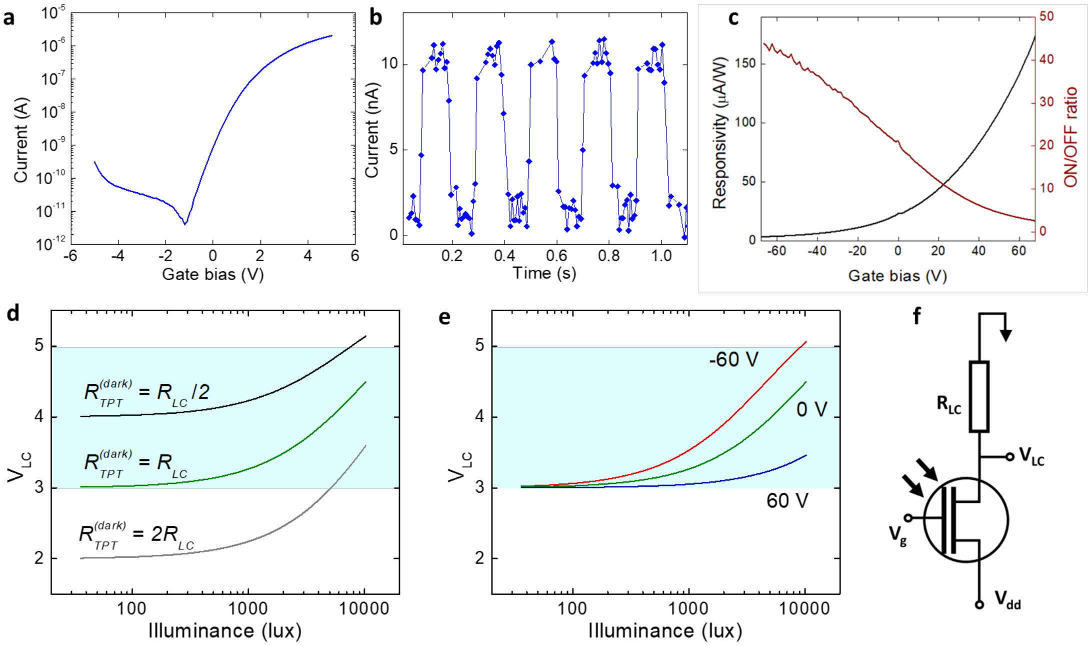

Supplementary Fig. 2 | Additional TPT characterization. a. The transfer curve of a VDWTF field effect transistor fabricated on a 50-nm Al_2O_3 back gate. The device is fabricated with an identical process to the TPT. The source-drain bias is 1 V. The slope leads to a derived transistor mobility of 25 $cm^2/(V\cdot s)$. The value is consistent with previously reported values of >10 $cm^2/(V\cdot s)$ (*Ref.*[33]). **b**. Photoresponse from a time sweep at 5-Hz chopping frequency. The observed rise and fall times are slower than the actual response time due to the lower sampling rate and the slow speed of the chopper edges as they cut through the laser beam diameter. The measurement conditions are identical to Fig. 2**e**. **c.** The extracted responsivity and ON/OFF ratio of a VDWTF FET fabricated on a silicon substrate with 300 nm SiO_2 body gate. V_{ds} = 1V. The photocurrent was tested under a thermal lamp illumination at 10^4 lux. **d**. The calculated voltage that is applied on the LC modulator (V_{LC}) with different TPT-LC resistance ratios. For the green curve, V_{LC} is below the nonlinear threshold at low intensities, but increases at higher incident intensity, which enables a strong nonlinearity. The gray curve does not enter the nonlinear region until very high intensities. The black curve is already in the middle of the threshold at low intensities, producing lower transmittance even under weak illumination. Gate tuning can change $R_{TPT}^{(dark)}$ to achieve a better resistance match, which leads to a larger V_{LC} shift across the threshold voltage range, leading to a stronger nonlinearity. **e**. V_{LC} under different gate biases (V_g). We assumed matched resistances ($R_{TPT}^{(dark)} = R_{LC}$) with different V_g. V_g = -60 V decreases the dark current, hence creating a larger ON/OFF ratio and larger V_{LC} shift across the threshold voltage range (highlighted in blue) under illumination. **f**. The equivalent circuit used to model the LC voltage in **d** and **e**, with V_{dd} = 6 V for both cases.

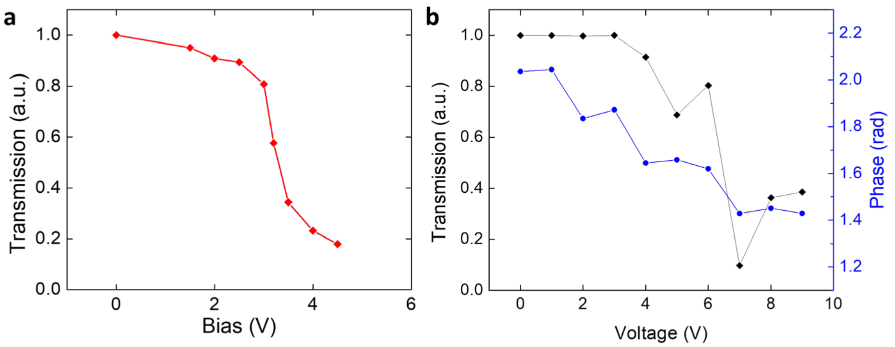

Supplementary Fig. 3 | LC modulation properties. a. Measured amplitude modulation of weak thermal lamp light with pure LC. The transmission is normalized by its value at 0 V. **b**. Extracted amplitude and phase modulation under weak light illumination. Data from a TPT-LC device different from the main device reported in the main paper. The device also shows a lower threshold voltage due to variations in TPT resistance as a result of process variation.

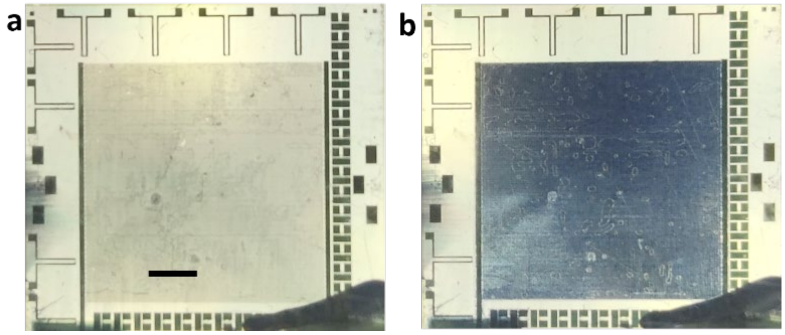

Supplementary Fig. 4 | Yield and uniformity characterization of the optoelectronic neuron array. The whole array stays below the LC modulator threshold at V_{dd} = 6 V (**a**) (higher than the LC threshold at 3V) but goes beyond the threshold at V_{dd} = 14 V (**b**), indicating no short-circuit or open-circuit TPT pixels.

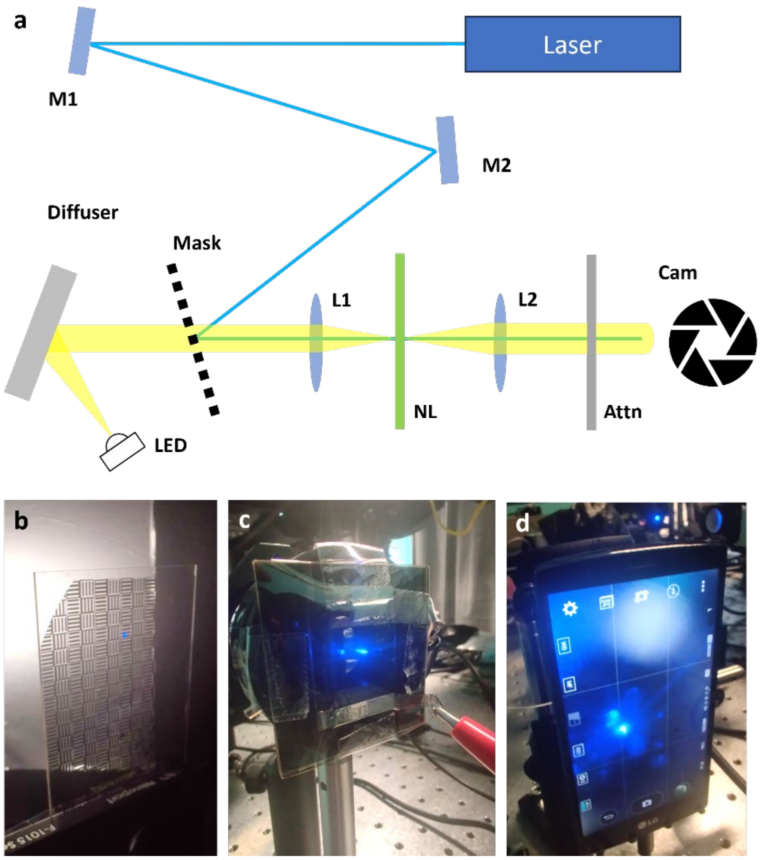

Supplementary Fig. 5 | The optical setup for the test. a. The schematic of the optical setup. The optical path of white light is highlighted in yellow, and the 473-nm laser path is highlighted in blue. The LED produces up to 1-W illumination that is diffusely reflected by the unpolished aluminum foil as the diffuser. The reflected light transmits through a patterned gold mask used as the object. Then, the pattern is imaged by lens L1 to the image plane that overlaps with the nonlinear layer NL. Lastly, the light passes through L2, which works as a magnifying system to facilitate better capture of the image at the smartphone camera. The laser beam is reflected by mirrors M1 and M2 to the metal mask surface. We tune M1, M2, and mask angles to collimate the laser beam with the white light path. **b**. A zoomed-in look at the metal mask. We remove the mask during uniformity measurements, so a relatively uniform white background is directly projected on the NL layer for transmission measurements. **c**. The packaged optoelectronic neuron array sandwiched by two orthogonal polarizers. It is connected to the voltage supply with the wires and mounted on an iris, which helps align the center of the device region to the optical path. **d**. The LG G4 smartphone used to capture the image. The image capture is set on the manual mode unless otherwise specified, with the following parameters: camera model: LGLS991, resolution = 72 dpi, bit depth = 24, color representation = sRGB, f/1.8, exposure time = 125 ms, manually set focal length = 4 mm, ISO-800, and digital zoom = 4.6.

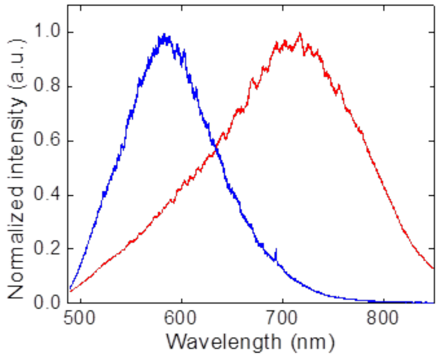

Supplementary Fig. 6 | Spectra of broadband incoherent light sources. Blue: the LED spectrum, with a center wavelength of 582nm. Red: the thermal lamp spectrum at the power level applied during the measurement, with the center wavelength at 713 nm.

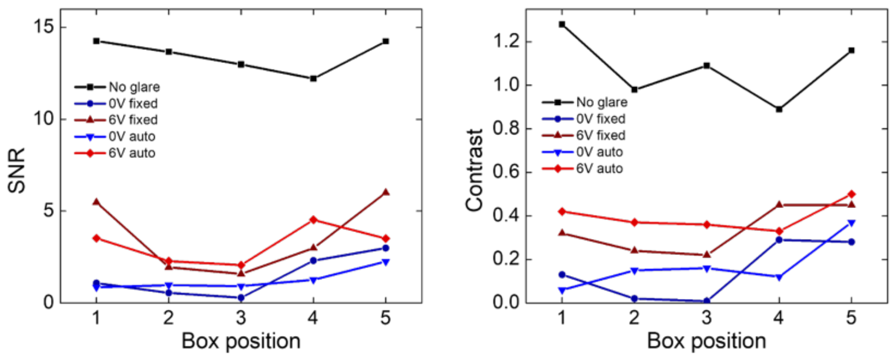

Supplementary Fig. 7 | SNR and contrast values evaluated at the boxes shown in Fig. 4 of the main text (with white rectangles).

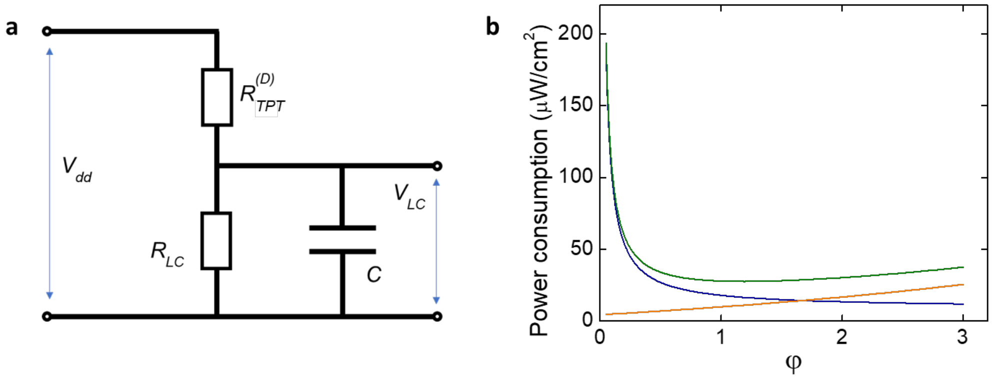

Supplementary Fig. 8 | Modeling and optimization of the optoelectronic neuron. a. The equivalent circuit for frequency response modeling. **b**. Estimation of the power consumption per unit area at different φ values. Blue: electrical power consumption; orange: photon loss; green: total power consumption. $R_{TPT}^{(D)}$ ~10 $G\Omega$ is the resistance of the MoS_2 channel in darkness, which was deduced from Eq. S1 from experimental data. The DC resistance of the LC layer, together with the LC alignment layers between the two ITO electrodes, is measured to be around 10 $G\Omega$ per 100 μm $\times$ 100 μm pixel with LC samples with identical fabrication process but with no TPT layers. The capacitance of the liquid crystal layer and its alignment layer (C) is calculated from experimental relative permittivity and device geometry parameters to be 71 fF/pixel.

Supplementary Table 1 | Comparison of nonlinear optical materials for broadband incoherent nonlinearity at low power threshold.*

Mechanism	Ref	Wavelength	Intensity Threshold	NL contrast	Speed	Max *T*	Broadband Incoherent Light
SHG	[20]	800 nm	10^{12} W/cm^2	80% (in SHG efficiency)	27 fs (pulse width limited)	close to unity	N
	[S1]	800 nm	10^{11} W/cm^2	60% (in SHG efficiency)	125 fs (pulse width limited)	close to unity	N
Nonlinear Kerr	[25]	1.5 um	10^4-10^5 W/cm^2	40% (by focused beam spot intensity)	~0.1 ns	N.A.	N.A.
Auger	[S2]	785 nm	10^{11} W/cm^2	2% (scattering)	80 ps	N.A.	N.A.
Saturable absorption	[24]	~1550 nm	266 MW/cm^2	64.4% to 65.7%	~100 fs	65.7%	N
Photo-chromic	[27]	405 nm	100 mW/cm^2	60% to 10%	> 1s	60%	Y
	[S3]	450 nm	10^7 W/cm^2	10%	10 us	N.A.	N.A.
Photo-refractive MQW	[32]	830-850 nm	100-300 mW/cm^2	3% (diffraction efficiency)	3 us	10%	N
	[33]	850 nm	10 kW/cm^2	0.1% (diffraction efficiency)	2 ns	19%	N
	[34]^	442 nm	100 uW/cm^2	0.1% (diffraction efficiency)	1 ms	N.A.	N
Photo-refractive doped LC	[S4]#	460 nm (W) 632 nm (R)	0.8 mW/cm^2	12% (diffraction efficiency)	6 ms	30% for writing beam	N
	[S5]$	488 nm	1 mW/cm^2	0.1% to < 0.0001%	40 ms (in power range excluding thermal effect)	0.1%	N.A.
	[31]%	488 nm	25 mW/cm^2	Transmittance 57% to 20%	80 s	57%	N.A.
	[S6]	1550 nm	3 W/cm^2	20% to 0.1%	3 ms at 300 W/cm^2; 40 s exposure applied during 3W/cm^2 test	20%	N.A.
Other photo-refractive	[38]	780 nm	2 W/cm^2	Reflectance <2% to 30%	2-5 ms	27% (reflection)	N.A.
TPT-LC Neurons	This work	473-650 nm and white light	0.1-10 mW/cm^2 ~56 μW/cm^2 (modeling)	70% contrast (ideally > 99%)	< 10 ms (limited by LCD, state-of-the-art at ~1 ms, > 3 kHz with faster modulators)	> 70%	Y

*Major limitations are highlighted in crimson. For the column of whether the technology is available for broadband incoherent light, 'N' means it is impractical because the mechanism either needs coherence or the threshold is too high to achieve with practical broadband incoherent sources. 'N.A.' means it could be possible but not demonstrated in the work. 'Y' means demonstrated to be valid. The references with 'S' refer to the supplementary references. Our optoelectronic neurons represent a unique technology that achieves the combined performance metrics (nonlinear threshold, speed, maximum transmission, etc.) necessary for processing broadband incoherent light captured by a camera lens under ambient illumination conditions, which other systems cannot achieve.

^ Operates at 77K.
# Tilted, good performance relies on lateral charge building up.
$ Thermal effect involved; non-thermal process relies on an attenuation-induced gradient.
% Disorder-induced diffraction fringes in glare reduction.

Supplementary Table 2 | Comparison of possible TPT material candidates.*

Material	Carrier mobility ($cm^2/V/s$)	Compatible to lithography	Tolerate thermal process	Need on-chip thermal process	Broadband response	Integration on other structures
Organics	Typically < 1	N	N	N	Y	Y
LTPS	>100	Y	Y	Y	Poor	Y
IGZO	10-100	Y	Y	Y	UV only	Y
Silicon on sapphire	~1000	Y	Y	N	Poor, ~50-time stronger absorption at 400 nm than 700 nm	N
III-V semi-conductors	>1000 (electrons)	Y	Y	Y	Y	Hard
VDWTF	10-30	Y	Y	N (if transfer)	Y	Y

*There are multiple material constraints set by the device structure, functionality, and fabrication process. The ideal material for the TPT in our design should have sufficiently high mobility to operate above 1 kHz, with a unique combination of transparency and optical response. It should also tolerate lithography and thermal processes after the thin film deposition, which is essential for making the SU-8, ITO, and LC structures on the top. The MoS_2 VDWTFs satisfy the fabrication processing compatibility that plagues many other candidate materials, and provide sufficient device performance for our reported application, which makes it an excellent choice for the application in consideration.

Supplementary Movie:

Movie captured by the cellphone at different exposure conditions with and without the intelligent glare reduction. The laser beam was chopped manually to create a dynamic glare.

Supplementary Note 1.

We first set a few basic parameters of the TPT-LC system with the experimental data. Material level optimizations may significantly improve the device performance beyond the calculated results based on our experimental parameters, which will not be discussed.

We tested TPT-free, large-area LC modulators with identical process flow to our optoelectronic neurons. At the threshold voltage V_{th} = 3 V, the current density is J = 3 μA/cm^2. Next, we tested the TPT-LC stack and observed an averaged V_{dd} = 6 V for the LC layer to enter the threshold. As a result, we can derive the average TPT resistance at a weak intensity from:

$$V_{LC} = \frac{R_{LC}}{R_{TPT}^{(D)} + R_{LC}} V_{dd} \qquad \text{(Eq. S1)}$$

The LC resistance per pixel (100 μm by 100 μm for our experimental devices) is R_{LC} = 3 V / 3 μA × $10^4 = 10\ G\Omega$. The dark resistance of the square MoS_2 channel is then $R_{TPT}^{(D)} = 10\ G\Omega$.

The capacitance of the liquid crystal is calculated with the permittivity of 5CB ($\varepsilon_r \approx 18$[1]) and PVA ($\varepsilon_r \approx 2$, slightly varies depending on sample preparation conditions[2]), with the thickness of PVA around 1 μm for both the upper and lower LC alignment layers. The ultimate capacitance is majorly limited by the lower-permittivity PVA layers and is around 0.71 nF/cm^2.

The equivalent RC circuit for a TPT-LC pixel is shown in Supplementary Fig. 7**a**. The LC driving voltage is related to the frequency following the equation:

$$V_{LC} = V_{dd} \frac{\frac{R_{LC}}{1+j\omega C R_{LC}}}{R_{TPT}^{(D)} + \frac{R_{LC}}{1+j\omega C R_{LC}}} = V_{dd} \frac{R_{LC}}{R_{TPT}^{(D)} + R_{LC}} \frac{1}{1+j\omega C \frac{R_{LC} R_{TPT}^{(D)}}{R_{LC}+R_{TPT}^{(D)}}} \qquad \text{(Eq. S2)}$$

We applied the dark resistance of the TPT, $R_{TPT}^{(D)}$, above since it gives the lower bound of the cutoff frequency compared with the light resistance. The 3dB cutoff frequency is then:

$$f_{3dB} = \frac{1}{2\pi} \left[C \frac{R_{LC} R_{TPT}^{(D)}}{R_{LC}+R_{TPT}^{(D)}} \right]^{-1} \qquad \text{(Eq. S3)}$$

Take the experimental values of our devices, $f_{3dB} = 452$ Hz. This RC delay, together with the intrinsic response time of LC molecules, limits the device response to a few hundred hertz. At the same time, the TPT layer can operate beyond kHz frequencies, and can be heterogeneously integrated with other types of modulators for higher operation speed.

Next, we discuss the scaling laws of the device. Since the two sources of power consumption, the photon absorption and the electrical power consumption, are both fixed with the total array area, increasing the number of pixels per area (N) will result in a reduction in the power consumption per activation proportional to N^{-1}. On the other hand, the product of $C \cdot R_{LC}$ remains constant during the scaling down, so the speed of the device remains constant as long as the TPT resistance increases accordingly. The TPT resistance can be well controlled by orders of magnitudes with changed channel geometry, annealing, and electrostatic gating for the VDWTF phototransistors. Considering the thickness of the LC modulation layer, edge effects of electric fields between parallel capacitors will become nontrivial when the pixel size is below 5 μm for

the current design. However, it is possible to reduce the LC thickness to sub-micrometers, so that the pixel size can go below 1 μm (ref [64])**Error! Bookmark not defined.**. Here we estimate the ultimate power consumption per pixel based on a 5-μm device size. This avoids imprecise estimations when the LC cell resistance changes with a thinner LC layer thickness.

The static electrical power consumption of the optoelectronic neurons is:

$$P_{elec} = V_{dd} I_{total} = \frac{R_{TPT}^{(D)} + R_{LC}}{R_{LC}} V_{th} I_{total} \qquad \text{(Eq. S4)}$$

The dynamic charging effect consumes a varied power depending on the input intensity change. Suppose the intensity changes are perfectly balanced in total power over all pixels. In that case, the dynamic charging is balanced on the common electrodes, contributing to no additional power consumption. On the contrary, if the whole array is suddenly uniformly illuminated, the dynamic charging cannot be balanced and consumes the energy $W_{dynamic}$ per charging:

$$W_{dynamic} = V_{dd} I_{charging} \Delta t = V_{dd} \Delta Q = C V_{dd} \Delta V_{LC} \qquad \text{(Eq. S5)}$$

ΔV_{LC} ~ 2 V is the voltage change to switch the LC from ON to OFF in our unoptimized LC modulators. Considering the worst case of uniform charging and discharging at the operation frequency f = 100 Hz, this leads to the maximum possible dynamic charging power:

$$P_{dynamic}^{(max)} = \frac{1}{2} C V_{dd} \Delta V_{LC} f \qquad \text{(Eq. S6)}$$

The factor of $\frac{1}{2}$ comes from charging and discharging per alternate cycles at 100 Hz. The value is 0.4 μW/cm^2 for our experimental device, which is much smaller than the static power consumption of 18 μW/cm^2 (Eq. S4) with the worst-case estimation.

Statistically, the dynamic charging power is proportional to the fluctuation of the total charging current $I_{charging}$ of all the pixels. Suppose the illumination on N_{pix} pixels are independent and fluctuate randomly, the total current fluctuation scales with $1/\sqrt{N_{pix}}$. Consequently, the mean dynamic charging power with N_{pix} pixels in the whole array is:

$$P_{dynamic}^{(mean)} = \frac{C V_{dd} \Delta V_{LC} f}{2\sqrt{N_{pix}}} \qquad \text{(Eq. S7)}$$

For our optoelectronic neuron array with 10,000 pixels, the value is 4 nW/cm^2 and negligible compared to the static electrical power consumption.

As discussed in the paper, the modulation from a high-transmission state to a low-transmission state can be achieved in $\Delta V_{LC} = 0.3\ V$ with optimized LC modulator structures. This can help us reduce the incidental intensity to trigger optoelectronic nonlinearity. The voltage change in LC is related to the TPT resistance change by:

$$\Delta V_{LC} = \left(\frac{R_{LC}}{R_{TPT}^{(L)} + R_{LC}} - \frac{R_{LC}}{R_{TPT}^{(D)} + R_{LC}} \right) V_{dd} = \left(\frac{R_{TPT}^{(D)} + R_{LC}}{R_{TPT}^{(L)} + R_{LC}} - 1 \right) V_{th} \qquad \text{(Eq. S8)}$$

The TPT resistances at different intensity levels are experimentally available with photocurrent measurements at the wavelength of 633 nm, as shown in Fig. 2**d**. We assume a linear relation between the total channel current I_{ds} and the incident power P_{inc}:

$$I_{ds}^{(L)} = (1 + \alpha P_{inc}) I_{ds}^{(D)} \qquad \text{(Eq. S9)}$$

We adopt a conservative estimation of weak-light photoresponse, assuming that the responsivity is constant below our lowest experimental illumination of 0.7 mW/cm^2. The assumption leads to a lower bound of the responsivity factor α =4.46, with P_{inc} having a unit of mW/cm^2. The actual responsivity at lower optical powers may exceed the fitted value, as defect saturation effects are much weaker under lower power illuminations. As a result, the presented estimation provides an upper bound of the necessary optical power for producing sufficient nonlinearity.

Take together Eq. S8 and Eq. S9, we have:

$$(1 + \alpha P_{inc})^{-1} = (1 + \gamma)^{-1} - \frac{\gamma\varphi}{1+\gamma} \qquad \text{(Eq. S10)}$$

$\gamma = \frac{\Delta V_{LC}}{V_{th}}$ and $\varphi = \frac{R_{LC}}{R_{PD}^{(D)}}$ are dimensionless parameters for brevity.

Consequently, the minimum necessary intensity is given by:

$$P_{inc} = \frac{\gamma(1+\varphi)}{\alpha(1-\gamma\varphi)} \quad \text{(in mW/cm}^2\text{)} \qquad \text{(Eq. S11)}$$

Taken the estimated parameter from our experimental devices, $\gamma = \frac{2\,V}{3\,V} = 0.67$ and φ =1, we have $P_{inc} = 0.91$ mW/cm^2. The threshold matches in its order of magnitude with the experimental device when operated at 8 V bias. For the potentially optimized devices with $\gamma = \frac{0.3\,V}{3\,V} = 0.1$ (typical nematic LC parameters taken from ref [59]) and $\varphi = 1.19$ (from the optimized device with minimized total power consumption, see later discussions), we would have $P_{inc} = 56$ μW/cm^2, much smaller than the unoptimized devices.

The total power consumption of the ideal device is:

$$P_{total} = \frac{1+\varphi}{\varphi} V_{th} I_{total} + A \frac{\gamma(1+\varphi)}{\alpha(1-\gamma\varphi)} \qquad \text{(Eq. S12)}$$

A = 0.2 is the photon loss through the TPT layer. Picking a larger φ value favors a smaller electrical power consumption, as a smaller V_{dd} is necessary to drive the device. However, it results in a larger optical intensity to create a larger TPT resistance change that shuts off the LC modulator. Supplementary Fig. 6**c** plots the power consumptions from photon loss and current flow. The minimum power consumption is 27.7 μW/cm^2 at $\varphi = 1.19$. Considering a scaled-down pixel size of 5 μm, the power consumption is 6.9 pW/pixel. Following Eq. S5 and S7, with $N_{pix} = 4 \times 10^6$ at 1 cm^2 total array area, $P_{dynamic}^{(max)} = 55$ nW/cm^2, and $P_{dynamic}^{(mean)} = 28$ pW/cm^2, which are much smaller than the static power. Hence, the dynamic charging power is again negligible. Finally, with an operation frequency of 100 Hz, the device can perform a nonlinear optical calculation at 69 fJ per activation.

Supplementary References:

1 Mori K, Tamaki Y, Obara M, et al. Second-harmonic generation of femtosecond high-intensity Ti: sapphire laser pulses. *Journal of Applied Physics*, **83**, 2915-2919, (1998).

2 Huang G J, Cheng H Y, Tang Y L, et al. Transient Super-/Sub-Linear Nonlinearities in Silicon Nanostructures. *Advanced Optical Materials*, **10**, 2101711, (2022).

3 Tokunaga A, Uriarte L M, Mutoh K, et al. Photochromic reaction by red light via triplet fusion upconversion. *Journal of the American Chemical Society*, **141**, 17744-17753, (2019).

4 Li X, Li Y, Xiang Y, et al. Highly photorefractive hybrid liquid crystal device for a video-rate holographic display. *Optics Express*, **24**, 8824-8831, (2016).

5 Khoo I C, Wood M V, Shih M Y, et al. Extremely nonlinear photosensitive liquid crystals for image sensing and sensor protection. *Optics Express*, **4**, 432-442, (1999).

6 Khoo I C, Park J H, Liou J. All-optical switching of continuous wave, microsecond lasers with a dye-doped nematic liquid crystal. *Applied Physics Letters*, **90**, (2007).

7 Kempaiah R, Liu Y, Nie Z, *et al*. Giant soft-memory in liquid crystal nanocomposites. *Applied Physics Letters*, **108**, 083105, (2016).

8 Reddy P L, Deshmukh K, Chidambaram K, *et al*. Dielectric properties of polyvinyl alcohol (PVA) nanocomposites filled with green synthesized zinc sulphide (ZnS) nanoparticles. *Journal of Materials Science: Materials in Electronics*, **30**, 4676-4687, (2019).
